# Assistive Products and Technology to Facilitate Activities and Participation for Children with Disabilities

**DOI:** 10.3390/ijerph20032086

**Published:** 2023-01-23

**Authors:** Johanne Mensah-Gourmel, Margot Thépot, Jan Willem Gorter, Maxime Bourgain, Christèle Kandalaft, Alain Chatelin, Guy Letellier, Sylvain Brochard, Christelle Pons

**Affiliations:** 1 Physical Medecine and Rehabilitation Department, Centre Hospitalier Régional Universitaire Brest, 29200 Brest, France; 2 Laboratoire de Traitement de l’information Médicale (LaTIM), Inserm U1101, Université Bretagne Occidentale, 29200 Brest, France; 3 Pediatric Rehabilitation Department, Fondation Ildys, 29200 Brest, France; 4 Physical Medecine and Rehabilitation Department, Assistance Publique-Hôpitaux de Marseille, 13005 Marseille, France; 5 CanChild Centre for Childhood Disability Research, Department of Pediatrics, McMaster University, Hamilton, ON L8S 1C7, Canada; 6 Department of Rehabilitation, Physical Therapy Science and Sports, University Medical Center Utrecht, Wilhelmina Children’s Hospital, 3584 EA Utrecht, The Netherlands; 7 Center of Excellence for Rehabilitation Medicine, UMC Utrecht Brain Center, University Medical Center Utrecht and De Hoogstraat Rehabilitation, 3583 TM Utrecht, The Netherlands; 8 Arts et Métiers Institute of Technology, Université Sorbonne Paris Nord, Institut de Biomécanique Humaine Georges Charpak, 75013 Paris, France; 9 EPF Graduate School of Engineering, 94230 Cachan, France; 10 Independent Researcher, 75013 Paris, France; 11 Fondation Paralysie Cérébrale, 75013 Paris, France; 12 Pediatric Rehabilitation Hospital, Etablissement de Santé pour Enfants et Adolescents de la région Nantaise—APF France Handicap, 44200 Nantes, France

**Keywords:** assistive products and technology, survey, needs, children, daily life, participation

## Abstract

We aimed to identify activity limitations and participation restrictions encountered by children and youth with disabilities for which assistive products and technology could be helpful. We used a convergent, parallel, mixed-methods design involving a nationwide, French survey composed of closed questions (quantitative) and open questions (qualitative) that enlightened the quantitative data. A total of 1055 responses were received, and 962 included: 92 from children and youth with disabilities, 493 from relatives and 377 from professionals. Difficulties frequently checked and described in detail were participation in recreational activities, leaving the house and traveling, participating in a group, and getting ready. Transversal explanations for difficulties were spontaneously provided (e.g., lack of accessibility and mobility). Solutions proposed included personal assistive devices to facilitate home life, high-tech devices, devices to compensate for impaired body functions, and adaptation of the familiar environment and daily activities. Few public solutions were proposed. The necessity of human assistance was emphasized. The mixed-methods design and involvement of different stakeholders identified common, macroscopic trends in difficulties encountered and desired solutions. Products and technology are required in the following domains: the familiar environment, accessibility and mobility, sports and leisure, high-technology, and family support. We provide suggestions to facilitate the development of innovative solutions.

## 1. Introduction

Children and youth acquire important skills through diverse life situations at home and school, and in leisure activities with family and friends. These skills help them to become autonomous and prepare for their future [1,2,3]. However, children and youth with disabilities are excluded from many such situations [4,5]. They experience activity limitations and participation restrictions as defined by the International Classification of Functioning, Disability, and Health (ICF) framework [5,6]. Facilitating their independence in performing the tasks required for involvement in life situations is key to improving both their participation and their development [7,8], and therefore, their health and wellbeing.

Environmental factors can be either facilitators or barriers to participation [7,9]. Identification of these factors is essential to overcome difficulties and facilitate involvement in life situations [2], taking into account the specific and growth-related needs of children [5]. Environmental factors include assistive products and technology [6], defined in the ICF as any products, instruments, equipment, or technology that are adapted or specifically designed to prevent, compensate for, reduce, or neutralize disability [5,10]. As stated by the United Nations International Children’s Emergency Fund (UNICEF), assistive products and technology are expected to give children and youth with disabilities “the opportunity to flourish as others might” [5].

Although a variety of assistive products and technologies exist, many needs are still unmet, partly because of difficulties accessing existing solutions [6,11]. Some products and technologies are designed on an individual basis, for example by parents or therapists, to fulfill specific, individual needs. Improving accessibility to existing assistive products and technology designed for large scale use is a current goal of the World Health Organization (WHO) and UNICEF [5,12], given that only 1 in 10 people in need actually have access to some assistive products. In parallel, improving the adoption of existing and available assistive technologies is a crucial issue [13]. For instance, in France, 30 to 40% of assistive devices are abandoned during the first year. Furthermore, many currently unmet needs could also be fulfilled by assistive products and technologies that have not yet been conceived [8,12]. 

The needs of children and youth with disabilities are not latent but explicit, and directly relate to difficulties in specific life situations [11]. Therefore, collecting the needs of many users with diverse profiles, including children and youth with disabilities, families, and professionals, constitutes the first step towards the development of pertinent solutions, as well as a prioritization of the development of future solutions. We thus designed and widely disseminated a survey with the purpose of prioritizing needs and solutions for diverse profiles of children and youth, in accordance with the WHO objectives for accessibility [5] and scaling up of health innovation.

The aim of this study was to (i) identify the most frequent activity limitations and participation restrictions for which assistive products and technology may be useful for children and youth with disabilities, and (ii) highlight macroscopic trends in difficulties encountered and desired assistive products and technology [6]. 

We hypothesized that certain life situations would be associated with specific difficulties, especially in unfamiliar environments or life situations. We also hypothesized that products and technology would be desired to facilitate these specific situations. We wished to use the results to propose suggestions to facilitate the development of innovative solutions.

## 2. Materials and Methods

This cross-sectional study used a convergent parallel mixed methods design [14] to collect quantitative data which would be enlightened by qualitative data. It was based on an online, open survey that followed the Strengthening the Reporting of Observational Studies in Epidemiology (STROBE) and the Checklist for Reporting Results of Internet E-surveys (CHERRIES) guidelines, and was conducted according to current French legislation [15]. In France, sociological studies are not included in studies involving humans (for instance, clinical trials), and French law indeed does not require ethical approval for such research protocols [15]. Data were anonymized, and responders could not be identified from the survey (no email or IP address), thus anonymity was guaranteed, and no ethical approval was required. The study was registered in Clinical Trials.gov: NCT05103930.

### 2.1. Consent

Children and youth or their carers, relatives, and professionals were informed of and accepted the use of their answers within the framework of a scientific collaboration.

### 2.2. Development of the Survey

The survey was developed by three physical medicine and rehabilitation physicians and two occupational therapists using the conceptual framework of the International Classification of Functioning, Disability and Health (ICF) [6]. It was pilot tested by two mothers of children with disabilities and an adult with a childhood-onset disability. Their comments were used to improve the survey.

**Figure 1:**
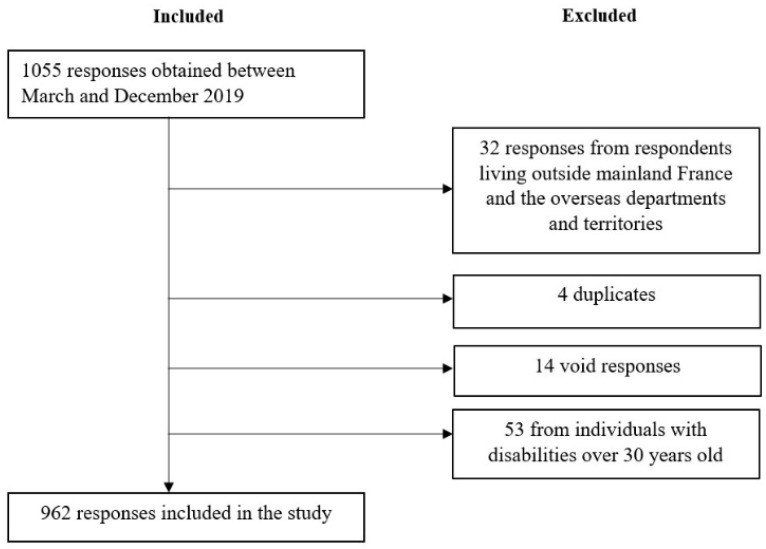
Flow chart.

The survey was created using google forms: bit.ly/innov-person, bit.ly/innov-relatives, bit.ly/innov-professional. Separate versions were developed for each of three participant profiles: children and youth with disabilities, their relatives, and professionals involved in the care of children and youth with disabilities. Exclusion criteria were (1) respondents who did not live in France, (2) duplicates, (3) void answers, and (4) adults with childhood-onset disabilities over 30 years of age (to avoid memory bias) (Figure 1). 

Each version was composed of 14 to 16 questions (6 pages). The first section described the study and the overall aim, and asked for epidemiological data. The second section was composed of a pre-established list (closed questions) of regular daily life situations; respondents were asked to select the situations which they felt were the most difficult for themselves/their relative/children and youth with disabilities (any number of items could be selected). All items were extracted from validated scales (MHAVIE/ABILHAND/ABILOCO KIDS/PEDI/ACTIVLIM). The list was proposed in chronological order to help the respondent to consider their own lives, if applicable. Two open questions were then asked. The first asked respondents to precisely describe the difficulties selected (termed difficulties) on regular weekdays. The second asked them to suggest ideas for technical solutions that could improve those situations (termed solutions). These questions were then repeated for weekends and holidays. The survey was designed to take less than 10 min to complete. Respondents could not be identified from the survey (no identity data; no email or IP address [16]). 

### 2.3. Data Collection

Convenience and snowball sampling were used to obtain data from participants in a wide range of situations between March and December 2019. The survey was promoted in France via the SFERHE (Société Francophone d’Etudes et de Recherche sur les Handicaps de l’Enfance), the EACD (European Academy of Childhood Disability), and at professional events (e.g., EACD 2019 Paris, France ; SOFMER (Société Française de Médecine Physique et Réadaptation) 2019 Bordeaux, France). A presentation leaflet was posted in hospitals and distributed on social media (Linkedin, Facebook, Twitter), and by e-mail. The survey was also sent to family and patient organizations (Paralysie cérébrale France (FFAIMC), Fondation Paralysie Cérébrale, Association Française contre les Myopathies, and professional networks (rare disease network). 

The first survey responses were used to propose some disability-related challenges to the students of the EPF engineering school during a disability-centered hackathon [17], without performing a full analysis of the answers. 

### 2.4. Data Analysis

Answers from children under five years of age were classified in the relative’s category of respondents, as we hypothesized that children of that age could not answer alone. 

Parallel analysis of datasets was performed. Quantitative data from responses to the closed questions were analyzed by descriptive analysis and the open questions by the framework method of qualitative analysis [18,19,20]. Inductive analysis from qualitative methods was chosen to bring out themes without a priori (and therefore, without relying on a pre-existing classification or on researchers’ ideas) [21,22,23]. Inductive analysis was performed at an explicit level [20]. Content analysis was performed to highlight the themes that emerged most frequently [20,21]. Each sentence that included different concepts was cut into phrases that corresponded to one idea. A harmonized codebook was developed by two researchers (JMG and MT). The codes were collated into categories and then themes. Triangulation was performed by regular debriefings with an external researcher (CP) [24].

## 3. Results

### 3.1. Participant Characteristics (Table 1, Tables S1–S3)

Table 1 summarizes epidemiological data (age, sex, region of residence, type of disability), Table S1 summarizes the pathologies of the respondents, Table S2 the relationship of the relatives, and Table S3 the type of professions. 

A total of 962 responses were included in the analysis:

- 92 children and youth with disabilities: 59.8% were ≤ 18 years old; 53.3% were females; 81.5% had a physical impairment; and 54.3% had a nervous system disease (23);
- 493 relatives: 77.5% were the mother, 81.5% of the people they responded for had a physical disability, 69.8% a mental disability, and 66.3% a cognitive disability; 36.7% had a nervous system disease;
- 377 professionals: 80.9% were females; 30.8% were rehabilitation professionals (not including physicians), 20.4% were physicians, and 12.5% were education professionals.

Responses were obtained from thirteen of the fourteen regions of France, with three regions more largely represented.

**Table 1:** Description of the respondents.

	Children and Youth with Disability (*n* = 92) ^1^	Relatives	Professionals (*n* = 377)
**Age (years)**		(*n* * = 488)	
Mean (SD)	17.1 (5.7)	46.6 (11.5)	41.3 (11.1)
Range (min-max)	[5–30]	[10–88]	[19–69]
Female	49	410	305
Male	43	78	72
**Region of residence**		(*n* * = 493)	
Auvergne, Rhône-Alpes	8 (8.7%)	41 (8.3%)	25 (6.6%)
Bourgogne, Franche-Comté	1 (1.1%)	15 (3%)	1 (0.3%)
Brittany	31 (33.7%)	181 (36.7%)	161 (42.7%)
Centre, Val de Loire	1 (1.1%)	12 (2.4%)	3 (0.8%)
Corsica	0 (0%)	0 (0%)	0 (0%)
Grand-Est	9 (9.8%)	25 (5.1%)	33 (8.8%)
Hauts de France	6 (6.5%)	29 (5.9%)	17 (4.5%)
Ile-de-France	11 (12.0%)	55 (11.2%)	62 (16.4%)
Normandy	0 (0%)	5 (1%)	4 (1.1%)
Nouvelle-Aquitaine	6 (6.5%)	32 (6.5%)	15 (4%)
Occitanie	7 (%)	25 (5.1%)	8 (2.1%)
Overseas	7 (7.6%)	1 (0.2%)	6 (1.6%)
Pays de la Loire	8 (8.7%)	51 (10.3%)	24 (6.4%)
Provence, Alpes, Côte-D’Azur	3 (3.3%)	21 (4.3%)	17 (4.5%)
No response	0 (0%)	0 (0%)	1 (0.3%)
**Type of disability**		(*n* = 493)	
**Cognitive**			
None	45 (48.9%)	111 (22.5%)	
Mild	10 (10.9%)	70 (14.2%)	
Moderate	15 (16.3%)	146 (29.6%)	
Severe	10 (10.9%)	111 (22.5%)	
I can’t tell/I don’t know	0 (0%)	16 (3.2%)	
No response	12 (13%)	39 (7.9%)	
**Mental**			
None	47 (51.1%)	106 (21.5%)	
Mild	13 (14.1%)	86 (17.4%)	
Moderate	8 (8.7%)	155 (31.4%)	
Severe	8 (8.7%)	103 (20.9%)	
I can’t tell/I don’t know	1 (1.1%)	10 (2%)	
No response	15 (16.3%)	33 (6.7%)	
**Physical**			
None	8 (8.7%)	61 (12.4%)	
Mild	12 (13.0%)	86 (17.4%)	
Moderate	30 (32.6%)	153 (31%)	
Severe	33 (35.9%)	163 (33.1%)	
I can’t tell/I don’t know	2 (2.2%)	7 (1.4%)	
No response	7 (7.6%)	21 (4.3%)	
**Sensory**			
None	45 (48.9%)	154 (31.2%)	
Mild	10 (10.9%)	104 (21.1%)	
Moderate	17 (18.5%)	129 (26.2%)	
Severe	4 (4.3%)	54 (11%)	
I can’t tell/I don’t know	2 (2.2%)	11 (2.2%)	
No response	14 (15.2%)	41 (8.3%)	
**Other**			
None	36 (39.1%)	58 (11.8%)	
Mild	2 (2.2%)	18 (3.7%)	
Moderate	6 (6.5%)	31 (6.3%)	
Severe	4 (4.3%)	26 (5.3%)	
I can’t tell/I don’t know	2 (2.2%)	29 (5.9%)	
No response	42 (45.7%)	332 (67.3%)	

^1^ *n* is the number of respondents for each item. * as answers from children under five years of age were classified in the relatives’ category, some data (age and sex) are missing.

### 3.2. Most Frequently Reported Difficulties (Table 2 and Table 3)

Table 2 and Table 3 summarize the results of the responses to the closed questions.

The weekday items most frequently selected by the whole sample as difficult were: “Doing sports, manual activities, or artistic activities”: 57.9%; “Leaving the house and traveling using any type of transport”: 57.4%; “Participating in a group, working, or studying”: 52.3%; and “getting ready” (getting dressed, brushing teeth, etc.): 50.9%. Children and youth with disabilities also frequently selected: “Participating in daily tasks at home”: 54.3%; and “Washing, body care”: 50%. Professionals frequently selected: “Participating in school trips, doing an internship”: 54%.

The items most frequently selected by the whole sample for weekends and holidays were: “Doing sports activities”: 54.8%; “Carrying out activities of daily life outside the home”: 50.6%; and “Going to play at a friend’s house or visiting somebody”: 44.5%. 

**Table 2:** Study results for difficulties during a typical day.

	Children and Youth with Disabilities (*n* = 92) ^1^	Relatives (*n* = 493)	Professionals (*n* = 377)	Total (*n* = 962)
Getting out of bed	30 (32.6%)	141 (28.6%)	86 (22.8%)	257 (26.7%)
Going to the toilet/bladder and bowel elimination	24 (26.1%)	210 (42.6%)	134 (35.5%)	368 (38.3%)
Organizing the day	30 (32.6%)	264 (53.5%)	182 (48.3%)	476 (48.5%)
Getting ready: e.g., getting dressed, brushing teeth, etc.	44 (47.8%)	298 (60.4%)	148 (39.3%)	590 (50.9%)
Eating breakfast	22 (23.9%)	134 (27.2%)	49 (13.0%)	205 (21.3%)
Leaving the house and traveling using any type of transport	35 (38.0%)	285 (57.8%)	232 (61.5%)	552 (57.4%)
Participating in a group, working, or studying	37 (40.2%)	285 (57.8%)	181 (48.0%)	503 (52.3%)
Using places or services like libraries or playgroups and getting there	23 (25.0%)	214 (43.4%)	161 (42.7%)	398 (41.4%)
Eating lunch	24 (26.1%)	146 (29.6%)	60 (15.9%)	230 (23.9%)
Doing sports, manual activities, or artistic activities	59 (64.1%)	314 (63.7%)	184 (48.8%)	557 (57.9%)
Eating a snack	15 (16.3%)	110 (22.3%)	44 (11.7%)	169 (17.6%)
Using a device like a computer, a video console, or a telephone, etc.	20 (21.7%)	149 (30.2%)	66 (17.5%)	235 (24.4%)
Participating in school trips, doing an internship	34 (37.0%)	229 (46.4%)	204 (54.1%)	467 (48.5%)
Playing (indoors or outdoors and alone or with friends)	23 (25.0%)	179 (36.3%)	90 (23.9%)	292 (30.4%)
Spending time with friends	22 (23.9%)	194 (39.4%)	120 (31.8%)	336 (34.9%)
Spending time with a boyfriend or a girlfriend (including intimacy)	20 (21.7%)	188 (38.1%)	178 (47.2%)	386 (40.1%)
Being a member of a sports club, an association, a religious community	26 (28.3%)	185 (37.5%)	116 (30.8%)	327 (34.0%)
Doing homework, studying at home	26 (28.3%)	216 (43.8%)	94 (24.9%)	336 (34.9%)
Washing, body care	46 (50.0%)	270 (54.8%)	155 (41.1%)	471 (49.0%)
Self-care (looking after glasses/orthosis, taking medications, etc.)	37 (40.2%)	277 (56.2%)	144 (38.2%)	458 (47.6%)
Participating in daily tasks at home	50 (54.3%)	277 (56.2%)	150 (39.8%)	477 (49.6%)
Chatting, reading	22 (23.9%)	210 (42.6%)	78 (20.7%)	310 (32.2%)
Having dinner with family	13 (14.1%)	89 (18.1%)	33 (8.8%)	135 (14.0%)
Going to bed	18 (19.6%)	140 (28.4%)	62 (16.4%)	220 (22.9%)
Sleeping	18 (19.6%)	104 (21.1%)	50 (13.3%)	172 (17.9%)
Other	13 (14.1%)	40 (8.1%)	25 (6.6%)	78 (8.1%)

^1^ *n* is the number of respondents who reported each life situation as difficult for children and youth with disabilities.

**Table 3:** Study results for difficulties during a day off (weekends, vacations).

	Children and Youth with Disabilities (*n* = 92) ^1^	Relatives (*n* = 493)	Professionals (*n* = 377)	Total (*n* = 962)
Going to play at a friend’s house or visiting somebody	25 (27.1%)	226 (45.8%)	177 (46.9%)	428 (44.5%)
Doing sports activities	52 (56.5%)	304 (61.7%)	171 (45.4%)	527 (54.8%)
Doing artistic, musical, or cultural activities	21 (22.8%)	203 (41.1%)	129 (34.2%)	353 (36.7%)
Going out in the countryside or town	28 (30.4%)	192 (38.9%)	142 (37.7%)	362 (37.6%)
Going to a leisure centre	14 (15.2%)	189 (38.3%)	109 (28.9%)	312 (32.4%)
Carrying out activities of daily life outside the home (e.g., at a friend’s house or elsewhere like a campsite or a hotel)	37 (40.2%)	257 (52.1%)	193 (51.2%)	487 (50.6%)
Going on holidays: journey (car, train, place, etc.)	38 (41.3%)	194 (39.4%)	181 (48.0%)	413 (42.9%)
Going on holidays: living in another place (flat, tent, etc.)	32 (34.8%)	186 (37.8%)	180 (47.7%)	398 (41.4%)
Going to the beach or swimming pool and doing water activities	32 (34.8%)	152 (30.8%)	105 (27.9%)	289 (30.0%)
Going to the mountains or playing outdoor games	30 (32.6%)	172 (34.9%)	108 (28.6%)	310 (32.2%)
Going on holiday abroad	29 (31.5%)	200 (40.6%)	159 (42.2%)	388 (40.3%)
Having fun, playing, relaxing	5 (5.4%)	106 (21.7%)	37 (9.8%)	148 (15.4%)
Going out with friends, going to parties, or evening events	16 (17.4%)	224 (45.4%)	117 (31.0%)	357 (37.1%)
Other	5(5.4%)	26 (5.3%)	12 (3.2%)	43 (4.5%)

^1^ *n* is the number of respondents who reported each life situation as difficult for children and youth with disabilities.

### 3.3. Macroscopic Trends in the Difficulties Reported

Analysis of the responses to the first open question generated 2430 difficulties codes. From the results, it emerged that participants particularly described difficulties (i) performing usual activities at home and (ii) participating in life situations outside the home. They also described difficulties that correspond to transversal factors that could induce or explain other difficulties described; for example, (iii) the lack of accessibility and mobility, and (iv) symptoms of the pathology, such as impaired mental functions (Table 4).

Within the familiar home environment, respondents detailed several situations which appeared to be particularly problematic and/or important, since they were often checked in the quantitative list. For example, dressing (often checked by all respondent-types): “fastening a bra for a quadriplegic teenager”; household chores and body care (often checked by those with a disability): “putting on a duvet cover for a visually impaired person”, “cutting nails”, eating: “cutting food” and “doing transfers”. 

It was striking that, for many respondents, difficulties increased outside of familiar, adapted environments. Difficulties with extracurricular school and leisure activities could be hard to overcome: “human assistance sometimes required during school trip”. Finding a “recreation center and day care that accepts children with disabilities”, with “trained professionals” was reported as difficult. For holidays and leisure activities, many respondents described difficulties performing regular tasks in unfamiliar environments (frequently checked in the closed questions). However, some activities, which were frequently selected as difficult in the closed questions, were less frequently described in the open responses. For example, respondents provided few specific details of difficulties relating to sport, other than limited access to “inclusive clubs” and “adapted equipment”. 

Respondents described transversal factors to explain difficulties with situations such as accessibility and mobility in both the urban and natural environments (which were frequently checked in the closed questions by all respondent-types): “public toilets are often dirty and do not have facilities to change an older child” or “accessibility to the beach, we need to carry our son on the sand”. Ensuring accessibility for all and in all conditions was an important issue; for example, if the lift at school breaks down: “sometimes places labeled as adapted are only partially adapted”. Issues with various means of mobility (wheelchair, adapted vehicle, etc.) were described, along with their conditions of use: “nowhere to recharge electric wheelchairs in public places, therefore autonomy is limited”.

Among the difficulties directly caused by the pathology, impaired mental functions were frequently reported as a transversal explanation for certain difficulties: “lag with children of the same age inducing frustration”. These factors limited both participation within a group and autonomy “decision dependency”. Impairment of voice and speech functions were frequently reported as impacting on communication, social relationships, and group participation: “Imprecise body language when communicating through sign language”. 

### 3.4. Macroscopic Trends in the Solutions Suggested

Analysis of the responses to the second open question generated 1037 solutions codes. Respondents (i) proposed personal assistive devices to facilitate usual activities (e.g., toileting, dressing, etc.); (ii) described the utility of high-technology; (iii) suggested equipment to compensate for impaired body functions and structure; (iv) described adaptation of the environment and daily life organization; and (v) highlighted the frequent need for human assistance (Table 5). 

Personal assistive products and technology to facilitate tasks within the home were very frequently described by all three categories of respondents, in line with the descriptions of frequent difficulties with life situations (Table 4). Some ideas related to pre-existing devices; others related to new solutions: “invent a Christmas tree that I could hang light-weight, magnetic decorations on”, “be able to mow the lawn with my electric wheelchair by fitting a mower on the back”. Suggestions were also made to improve existing devices and their format: for instance, respondents expected solutions to be practical, space-saving, and as inexpensive as possible; one other suggestion was for “a loan and/or rental system”.

High-tech solutions were proportionately more frequently suggested by children and youth with disabilities. They wanted software to facilitate mobility, communication, access to social networks or meeting groups, planning, and organization. Hardware needs related more to recreational tools: “adapted video game controller to play with friends”. “Robotization” and “home automation” were suggested to improve autonomy; “improvement of Google home (reliability) with better device control settings”.

Rehabilitation professionals provided more specific details of the needs for rehabilitation equipment and devices to compensate for impaired body functions and structures; e.g., “an upper limb prosthesis to facilitate the use of a musical instrument”. Several examples were suggested to compensate for a lack of executive functions: e.g., “a timer” and a “timetable with pictograms”.

Suggestions made by relatives frequently related to adaptations of daily life organization and the environment; e.g., “taking butter out of the fridge in advance to help make toast” and “respect a location for each object”.

The unavoidable need for “human assistance” was frequently mentioned, particularly by relatives. This highlighted the high level of family compensation for difficulties that technology does not yet solve, and the impact of the disability overall on the entire family.

## 4. Discussion

This study used an online survey to collect the points of view of a large sample of children and youth with disabilities, relatives, and professionals, and combined quantitative and qualitative data to reveal macroscopic trends in the difficulties encountered and solutions desired. 

### 4.1. Macroscopic Trends

Difficulties performing tasks involved in common life situations at home and in familiar environments were highlighted in both the closed and open questions by all categories of respondents. Facilitating smooth performance and independence in such tasks is essential [25]. The large number of suggestions for personal products and technology to relieve these difficulties confirmed our hypothesis that assistive devices can help to overcome some difficulties, that they are already used, but that knowledge of their existence, access, and availability should be improved. These findings are in accordance with WHO and UNICEF reports; but, moreover, the answers from this study also suggested that some solutions might be improved and that new innovative solutions are also required. The large number of answers gathered helps to draw macroscopic trends and highlight specific issues. For instance, children and youth with disabilities particularly suggested high-tech solutions they believed could help them. These results are important since the high-technology domain is little developed in the ICF. These findings suggest that an update of the sections of the ICF that relate to products and technology to facilitate amical and social relationships could be considered, including high-technology products such as digital applications, social media, and video games.

The responses to the qualitative questions showed that more difficulties were encountered in unfamiliar situations and environments than in familiar ones. Despite this, fewer solutions were suggested for these situations, and they were less precisely described. For example, participation in sports and leisure activities was frequently rated as difficult in the responses to closed questions, but relatively poorly described in the answers to the open questions. Participation in these domains is often low for people with disability; therefore, their lack of experience may have led to a lack of suggestions [3,4]. A major issue is the lack of financial support for assistive devices for sport [26]. There is therefore a large need for new, inexpensive solutions to facilitate access to sports and leisure. Another barrier to participation in leisure activities was accessibility. Respondents reported both direct issues relating to mobility and accessibility, such as available/adapted transport and infrastructures, and indirect issues such as limited access to culture, leisure, holidays, etc. Again, despite the high frequency of selection of these difficulties in the closed questions, and frequent descriptions in the first open question, relatively few solutions were proposed. Accessibility is a public issue; these results show that much work is needed for the development of public resources (as defined by the ICF), and not just personal products and technology, to facilitate participation for a large and diverse pool of users [5]. 

An important and unexpected result of this study was the lack of correspondence between the number of difficulties and the number of solutions that emerged: around three times fewer codes were generated for solutions than for difficulties. Therefore, the development of new solutions may be urgent. Specific methodologies are required to facilitate the emergence of ideas for desired solutions for children and youth with disabilities, especially (i) public solutions and (ii) solutions for little-experienced difficult life situations. When no solutions existed or were imagined, the responses clearly demonstrated how the family compensated, their capacity for adaptation and flexibility, as well as the impact of the disability on the entire family. 

### 4.2. Suggestions for the Development of Innovative Solutions

A comprehensive approach to difficulties is essential for the development of solutions. In high-income countries, even when assistive technologies are available, their adoption is not ensured–in France, 30 to 40% of assistive devices are abandoned during the first year [13]. A better understanding and definition of the need may facilitate the choice of the most relevant assistive device. To this end, the following suggestions may help to precisely describe the need. Firstly, the results highlighted the importance of seeking transversal factors that explain difficulties, as well as questioning both experienced difficulties and desired solutions, since suggestions may be related or unrelated to a remembered difficulty. Secondly, consideration of the points of view of children and youth with disabilities, their relatives, and professionals allows a complete picture to be drawn [25,27,28]. Thirdly, it emerged that difficulties were greater in unfamiliar situations and environments. This should be considered by solution providers: when designing a new solution, users should not only be staged within their familiar environment and activities, but also in new experiences. Fourthly, it is essential to develop public (not only personal) products and technology to remove barriers for as many people as possible. 

New methodologies that aim to facilitate collaboration and foster innovation, such as hackathons [8,29], living labs [30], user-centered designs, or design thinking [31], may be highly useful in this process. Collaborations should be pursued between all relevant parties for the development of new solutions that fulfill the expectations of a larger number of people, while considering the possible side effects of technologies. Those collaborations could be extended to stakeholders such as public policy-makers, architects, town planners, charities, and non-disabled persons. Lastly, marketed devices already exist for some of the desired solutions; improving dissemination of information must therefore be a priority. Specific processes, such as training courses, could also be put in place to facilitate the acceptance of new technologies and, therefore, their appropriate implementation [32].

### 4.3. Limits

The use of snowball sampling with social media prevented calculation of a response rate; however, it generated responses from a large number of persons with varied pathologies and functional situations. The proportion of children and youth with disabilities who responded was relatively small, likely due to their age and/or their degree of dependency. Future studies should use methods specifically designed for interviewing/questioning children and youth with disabilities [25]. The survey was custom made and was not validated. However, our approach was new, and to our knowledge, no validated tools for the evaluation of needs for innovation in assistive products currently exist. This survey was conducted in France to ensure consistency and to gather responses within a single medical-economic system; generalization outside France should be done with prudence.

## 5. Conclusions

Products and technology are required to facilitate life situations for children and youth with disabilities in the following domains: the familiar environment, accessibility and mobility, sports and leisure, high-technology, and family support. 

Processes should: (i) in addition to assessing difficulties and needs, seek transversal explanatory particularities such as impaired mental functions, or lack of accessibility and mobility, which can induce and explain the difficulties described; (ii) consider the points of view of the individuals concerned, their relatives, and professionals to build a complete picture; (iii) stage end-users in new experiences since difficulties increase outside familiar environments and situations; and (iv) develop public (rather than personal) products and technology to remove barriers for as many people as possible.

To increase activity and participation, society and solution providers need to create opportunities and give support to engage in activities and participate in rarely accessed situations and environments to explore difficulties and solutions.

## Figures and Tables

**Table 4:** Examples of experienced difficulties described in the responses to open questions (in bold style: responses from relatives; in italic style: responses from professionals; in regular style: responses from children and youth with disabilities).

**PERFORMING USUAL ACTIVITIES AT HOME**
*Dressing can be complicated, particularly for a tetraplegic woman, like putting on a bra (fastenings).*
Do my hair, make a pony tail.
Peel vegetables.
**It is hard for my son who is visually impaired to fasten his trousers or his jacket.**
*Making your bed requires a lot of concentration to put a duvet cover on properly (visually impaired).*
**My daughter can’t go to the toilet by herself, she can’t get up herself or transfer from her chair to the toilet, she can’t stand even with help.**
**Difficulty tidying the house/clearing the table, etc., because can’t carry and walk at the same time.**
**PARTICIPATING IN LIFE SITUATIONS OUTSIDE THE HOME**
*Very few activity clubs take children with disabilities.*
**Holiday camp: needs a trained helper and sensory equipment.**
**My son gets about on his bottom, which makes outdoor games with other children difficult.**
**For outings, my daughter can’t walk and we have to use a pushchair (too small for a wheelchair), which doesn’t allow her to integrate socially in a group of children.**
**My son loves horses but he can’t ride because he can’t sit, he spends his day in a molded seat.**
**School outings are not possible unless a parent goes along to help the child to get about (the school assistant for disabled children has to stay at school with the other children).**
Do team sports.
I can’t go to the cinema because the one in my town is not accessible. If I want to go, I have to go to another town which is quite far from mine.
**Sport is not possible, unless you have adapted equipment and advice.**
**Not disabled enough to do adapted sports, he has no place anywhere, and too disabled to do sport with “typical” children who only judge him on his results.**
*Difficult to set up art-type activities (e.g., painting) because tables and chairs are not adapted for all children, child poorly positioned.*
**We can’t go on holiday because we need to have an ambulance to travel lying down for trips longer than 1h.**
*Difficulty knowing where one is in town.*
*As an adapted physical activity teacher, I find that accessibility of equipment is a frequently encountered problem.*
**The fact that my son is severely incontinent is another problem for organizing long outings because he has many “incidents” and because of his age–24 years–it is very complicated to change him depending on the place.**
**My daughter can’t climb play structures with ropes, ladders, etc.**
**On holiday she loves swimming in a pool, but we have to carry her into the water, and it is really difficult to get her out!!! And one of us must be with her constantly in the water! Unfortunately, we have not been to a pool for 2 years because she is 16 and has put on a lot of weight!**
**LACK OF ACCESSIBILITY AND MOBILITY**
*Wheelchair accessibility is less good in our towns and especially our countryside, with stops young people from being fully autonomous.*
*Bus and tram stops are not all adapted for people in a wheelchair.*
**Putting a child in a car who doesn’t want to go in is difficult.**
Difficulty travelling by plane, being able to stay in my electric wheelchair and take a passenger place.
To travel, it is necessary to look for adapted housing (bedroom and bathroom), it’s like moving house bringing all my equipment (wheelchair, toilet chair, pump, and feeding tube, etc.).
The hardest thing for me is to go shopping by myself, take money out... cope with accessibility in town.
**When we go on holiday in a holiday house said to be adapted, we often have problems in the adaptations in the bathroom and toilets.**
**Foreign hotels sold as “adapted” with 5 to 10 stairs smoothed into a “wheelchair slope” (even after going to the agency to check!!).**
**Not all holiday camps have a lift and it is too costly for them to install one.**
*Carrying bags through an automatic entry door in a block of flats—the opening time is too short.*
*In France, we are way behind compared to other countries (particularly Northern countries) in accessibility people with disability, for both people with physical disabilities (little accessibility in hotels, campsites, access to public transport) and sensory impairments (blind, pavements and crossings adapted for all types of disability, access to public transport etc.).*
*Accessibility of public places, cluttered pavements, broken down lifts in schools, few accessible buses in establishments for young people because of high costs,* etc.
*All trips are complicated: accessibility of places, transportation of wheelchairs, moulded seats, feeding, potential separation from parents* etc.
*Going places with the Motilo, standing frame, and molded seat, it puts you off from the start...*
*Nowhere to recharge (electric wheelchair) in public places for example and limited autonomy.*
**For the helper, attaching the chair with straps is exhausting, especially as you get older.**
*Vehicles that are adapted for electric wheelchairs are too costly for most families.*
*Beaches are hardly accessible, some have a ramp but it doesn’t go to the edge of the water, and there is no specialized beach equipment (like a beach wheelchair).*
**SYMPTOMS OF THE PATHOLOGY**
**My son has lots of difficulties at school, following, staying concentrated, making friends etc. [...]. In general, his agitation, his emotions that are difficult to control and his high level of fatigability prevent him from doing most of the activities that he sees his classmates doing.**
*An autistic child in our class doesn’t talk, so it is impossible for him to discuss things.*
**Our child has difficulty planning and organizing tasks. He can’t do two tasks at the same time. He has big attentional difficulties. These difficulties have a severe impact on his schooling. He can’t write and listen at the same time. If there are two instructions, he forgets one.**
**My son cannot communicate verbally which has a huge impact on his social life and learning. He is totally isolated, despite the compensatory tools that have been set up (signing, communication software).**
**My child gets lost in public transport (he doesn’t know which direction to take the transport even when he is in the right one). He needs a GPS even when he is walking.**
**My daughter has a mild cognitive impairment, she has difficulty with mental arithmetic, which impacts on her daily life for example, she has difficulty giving change.**
**Our son walks, not well but sufficiently well, to run off and put himself in danger because he is unpredictable and has no sense of danger.**
**No decisional autonomy.**
**Time with friends ends in conflict because he can’t control his emotions. Family dinners are impossible.**

**Table 5:** Examples of desired solutions described in the responses to open questions (in bold style: responses from relatives; in italic style: responses from professionals; in regular style: responses from children and youth with disabilities).

**PERSONAL ASSISTIVE DEVICES TO FACILITATE USUAL ACTIVITIES**
For sleeping, I would like a blanket that does not stop me repositioning myself in my bed because of its weight, but that is thick enough to be warm.
A sort of automatic hoist.
I would need a robot that bends and straightens my legs at night just by pressing on a little contactor. That way I wouldn’t have to get my parents up.
**I tried to make the snap buttons brighter (fluorescent yellow).**
**An adjustable table with compartments to have everything at hand.**
**Inventing games that are not too childish for my daughter (as she is a teenager) but not too complicated.**
*Offer adjustable equipment (wheelchair, etc.) because some children grow quickly.*
*Equipment that is available is still very expensive and not very accessible for families.*
**We have just bought a toothpaste press to avoid half the tube going on the brush because of difficulty controlling the forces of fine movements.**
**In summary, innovations that would help us to avoid movements and postures that are bad for our health as carers (back, etc.).**
**We need an ultra-foldable means of transport to use when we go for a walk and our daughter doesn’t want to walk anymore and sits on the ground, she is too big for a pushchair and pushchairs are not at all practical for us (take space), she doesn’t need a wheelchair because she can walk.**
**UTILITY OF HIGH-TECHNOLOGY**
For writing a message on a smartphone with one hand, the creation of a more suitable application than voice recognition for example.
Adaptation of the PS4 gamepad. Because I can’t play it, I can’t play with my friends at the holiday club.
A small companion robot to refocus attention problems.
**So that she can use her phone or computer we made a stylus by joining several together. She puts it in her mouth to type on the screen. Nothing like that exists.**
**For homework, an application that suggests a schedule.**
*Pooling of equipment or possibility of finding equipment at lower cost on site or for occasional use (recycling equipment no longer in use, etc.). Create an official website that would list the places where equipment can be found.*
*Simultaneous translation of spoken language into sign language by smartphone could help.*
*For physical activities and sports, we are currently developing an application for visually impaired and blind people to enable them to sail. The application is coupled with a sensor placed on the sail to deliver useful and essential sound information for navigation.*
**Google home (but more reliable and regular than at present) if completed by well parameterized electronics to work equipment (bed, TV, telephone, etc.).**
**Contactor interface connections are a dream for toys, they should be sold as part of the standard range.**
**A free online software that will allow the child to learn to use a keyboard without looking, which reproduces the sound of the letter.**
*Inclusive events platform*
**EQUIPMENT TO COMPENSATE FOR IMPAIRED BODY FUNCTIONS AND STRUCTURE**
*Specifically oriented finger prosthesis and relieves the weight of the instrument.*
*We use pictograms, sign language to facilitate communication.*
**Installation of an epilepsy kit between the bed base and the mattress and a videophone in his room.**
**Connected watch with heart rate and SaO2 measurement that alerts the emergency services/parents in case of an anomaly.**
**DESCRIBED ADAPTATION OF THE ENVIRONMENT AND DAILY LIFE ORGANISATION**
My Mum set me up with a magnetic schedule so that I know what I’m supposed to be doing.
**We take out the butter earlier for the toast.**
**We have colored objects so he can see them.**
**We try to have as many games as possible stored at her height so that she can help herself, but it’s not always easy to find shelves with drawers that can be opened easily...**
**We have to make showering into a game by making bubbles to have breaks.**
*Organize their schoolbag for high school.*
**NEED FOR HUMAN ASSISTANCE**
**We (the parents) asked the town council for extra staff and I asked the sports club if I could accompany my daughter to an activity to assess the dangers and protect her if necessary.**
**Always needs personalized support to reassure, explain, avoid situations that are too anxiety-provoking.**
**He can’t visit people independently, so we always have to go with him.**
I need permanent help from an assistant.

## Data Availability

The full data associated with the paper are not publicly available but are available from the corresponding author on reasonable request.

## Supplementary Materials

The following supporting information can be downloaded at: https://www.mdpi.com/article/10.3390/ijerph20032086/s1, Table S1: Study results for the pathologies of the respondents or of the relative (s) of the respondents; Table S2: Study results for the type of relationship of the relatives; Table S3: Study results for the type of professions
