# Supplementary material for: Assistive Products and Technology to Facilitate Activities and Participation for Children with Disabilities
Source: Int J Environ Res Public Health. 2023 Jan 23;20(3):2086. doi: 10.3390/ijerph20032086 (PMC9915405; doi:10.3390/ijerph20032086)
Supplement: Supplementary file 1 [file ijerph-20-02086-s001.zip › ijerph-2150669-supplementary.pdf]

**Table S1**. Study results for the pathologies of the respondents or of the relative(s) of the respondents.

| | Children and Youth n=92 | Relatives n=493 |
|---|---|---|
| Certain conditions originating in the perinatal period | 1 (1.1%) | 4 (0.8%) |
| Infectious and parasitic diseases including: | 3 (3.3%) | 0 (0%) |
| - Poliomyelitis | 3 (3.3%) | |
| Injury: | 1 (1.1%) | 0 (0%) |
| - Traumatic amputation of leg | 1 (1.1%) | |
| Traumatic head injuries | 0 (0%) | 4 (0.8%) |
| Diseases of the circulatory system | 1 (1.1%) | 1 (0.2%) |
| Diseases of the eye and adnexa | 2 (2.2%) | 4 (0.8%) |
| Diseases of the ear and mastoid process | 2 (2.2%) | 3 (0.6%) |
| Diseases of the blood and blood-forming organs and certain disorders involving the immune mechanism including: | 6 (6.5%) | 42 (8.5%) |
| - Di George's syndrome | 4 (4.3%) | 42 (8.5%) |
| Diseases of the nervous system including: | 50 (54.3%) | 181 (36.7%) |
| -Spinal muscular atrophy | 4 (4.3%) | 13 (2.6%) |
| -Vascular syndromes of brain | | 11 (2.2%) |
| -Myopathies | 17 (18.5%) | 22 (4.5%) |
| -Cerebral palsy and other paralytic syndromes | 25 ( 27.2%) | 105 (21.3%) |
| Diseases of the musculoskeletal system and connective tissue | 2 (2.2%) | 1 (0%) |
| Endocrine, nutritional and metabolic diseases | 3 (3.3%) | 10 (2.0%) |
| Congenital malformations, deformations and chromosomal abnormalities including: | 9 (9.8%) | 128 (25.0%) |
| -Angelman syndrome | | 12 (2.4%) |
| -Williams syndrome | | 13 (2.6%) |
| -Trisomy | | 17 (3.4%) |
| Symptoms, signs and abnormal clinical and laboratory findings, not classified elsewhere | 0 (0%) | 2 (0.4%) |
| Genetic syndromes | 0 (0%) | 1 (0.2%) |
| Mental and behavioural disorders including: | 7 (7.6%) | 77 (15.6%) |
| -Autistic spectrum disorder | 4 (4.3%) | 39 (7.9%) |
| Neoplasms | 3 (3.3%) | 4 (0.8%) |
| Unclassifiable | 1 (1.1%) | 14 (2.84%) |
| No response | 1 (1.1%) | 17 (3.4%) |

**Table S2**. Study results for the type of relationship of the relatives.

| | |
|---|---|
| Mother | 378 (77.5%) |
| Father | 64 (13.1%) |
| Sister | 9 (1.8%) |
| Brother | 5 (1%) |
| Aunt/Uncle | 4 (0.8%) |
| Cousin | 1 (0.2%) |
| Grand parent | 17 (3.5%) |
| Friend | 3 (0.6%) |
| Other | 7 (1.4%) |

**Table S3**. Study results for the type of professions.

| | |
|---|---|
| Early childhood professional | 3 (0.8%) |
| Engineer | 2 (0.5%) |
| Healthcare worker | 29 (7.7%) |
| Physician | 77 (20.4%) |
| Recreational worker in an activity centre/sports centre | 4 (1.1%) |
| Rehabilitation specialist | 116 (30.8%) |
| Specialised educator | 25 (6.6%) |
| Teacher | 15 (4%) |
| Other | 106 (28.1%) |
